# Isolation of *Leptospira* serovar Pomona from a crested porcupine (*Hystrix cristata*, L., 1758)

**DOI:** 10.1002/vms3.308

**Published:** 2020-06-17

**Authors:** Giovanni Cilia, Fabrizio Bertelloni, Francesca Coppola, Barbara Turchi, Claudia Biliotti, Alessandro Poli, Francesca Parisi, Antonio Felicioli, Domenico Cerri, Filippo Fratini

**Affiliations:** ^1^ Department of Veterinary Sciences University of Pisa Pisa Italy; ^2^ CRASM “Semproniano” Grosseto Italy

**Keywords:** crested porcupine, isolation, *Leptospira*, MLST, zoonosis

## Abstract

Pathogenic *Leptospira* is widespread in rodents, the most studied *reservoir* and the main hosts involved in its transmission. In Italy, among rodents, *Hystrix cristata* (crested porcupine) is the largest species and it is distributed all over the country. In this paper, the isolation and characterization of pathogenic *Leptospira* spp. from the kidney of *H. cristata* is reported for the first time. During Autumn 2018, *Leptospira* detection by real‐time PCR and isolation were performed from kidneys of two died female porcupines (an adult and a porcupette). Only for porcupette kidney sample, real‐time PCR for pathogenic *Leptospira* tested positive. The isolated strain was identified as *Leptospira interrogans* serogroup Pomona serovar Pomona, using the three schemes of multilocus sequence typing. The results show that *H. cristata* could be a *Leptospira* host. The infection of serovars Pomona could be related to the habitat shared with wild boar, a typical *reservoir* host for this serovar.

## Graphical abstract

In this paper, the isolation and characterization of pathogenic *Leptospira* spp. from the kidney of a crested porcupine (*Hystrix cristata*) is reported for the first time. The isolated strain was identified as *Leptospira interrogans* serogroup Pomona serovar Pomona.

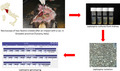

## 1 INTRODUCTION

Leptospirosis is a re‐emerging bacterial zoonosis (Chikeka & Dumler, 2015; Picardeau, 2017; Ruiz‐Fons, 2017) with a wide distribution in tropical, subtropical and temperate areas. The spreading of this disease is favoured by the presence of a large diversity of wild and domestic mammals with the function of *Leptospira reservoir* (Chikeka & Dumler, 2015; Faine, Adler, Bolin, & Perolat, 1999). Among asymptomatic maintenance hosts, localization of *Leptospira* occurs in the kidney, liver and in some animals, such as bovine or swine, also in the reproductive tract (Ellis, 2015). *Leptospira* localization in the kidney is the main route of shedding and environmental spread of this bacterium (Adler & de la Peña Moctezuma, 2010; Ellis, 2015). *Leptospira* epidemiology is strictly related to the presence of the maintenance hosts species (Cerri, Ebani, Fratini, Pinzauti, & Andreani, 2003). Recently, association between serovars and new wild and domestic species emerged, suggesting variation in leptospirosis epidemiology in both humans and animals (Tagliabue et al., 2016).

Rodents are considered among the most important *reservoir* of *Leptospira* (Adler & de la Peña Moctezuma, 2010). In different countries, some rodents, such as for *Apodemus* spp., *Bandicota* spp., *Delomys* spp., *Mus* spp., *Necromys* spp., *Oryzomys* spp., *Rattus* spp., *Thaptomys* spp., *Trinomys* spp. and also for *Myocastor coypus,* and *Hydrochaeris hydrochaeri*s were described as *Leptospira* maintenance hosts (Cortizo et al., 2015; Cosson et al., 2014; Fratini et al., 2015; Jorge et al., 2012; Krijger, Ahmed, Goris, Groot Koerkamp, & Meerburg, 2019; Levett, 2001; Michel et al., 2001; Moreno, Miraglia, Marvulo, et al., 2016; Vieira et al., 2019; Vieira, Pinto, & Lilenbaum, 2018).

Recently, crested porcupine (*Hystrix cristata*, L. 1758) sera collected in Italy resulted positive for serogroup Icterohaemorrhagiae, Australis and Pomona (Coppola, Cilia, et al., 2020). Furthermore, among porcupines (Rodentia; Hystricomorpha), *Leptospira* serovar Pomona, was isolated from the blood, urine and kidney of one North American porcupine, *Erethizon dorsatum* (Mitchell, Robertson, Corner, & Boulanger, 1966). Serological investigation on Malayan porcupine (*Hystrix brachyura*) evidenced antibodies against the serovars Javanica, Hurstbridge, Ballum, Celledoni and Hardjoprajitno (Siti‐Nurdyana, Bahaman, Sharma, Azlan, & Abdul Razak, 2016). Molecular analysis on the DNA extracted from urine of *Sphiggurus villosus* (orange‐spined hairy dwarf porcupine) also evidenced a renal *Leptospira* infection, despite the microscopic agglutination test (MAT) resulted negative (Fornazari, Langoni, Marson, Nóbrega, & Teixeira, 2018).

Italy is the only European country where the crested porcupine live in the wild as naturalized specie (Coppola, Dari, Vecchio, Scarselli & Felicioli, In press, Santini, 1980). Porcupine is the largest rodent of the Italian fauna and is widely distributed in mainland and it is also present in Sicily and Sardinia. (Loy et al., 2019; Mori, Sforzi, Bogliani, & Milanesi, 2018). Recently, Vecchio, Coppola, Scarselli, Giannini, and Felicioli (2018) report the presence of at least one free‐ranging crested porcupine in the Island of Elba using camera‐trapping rising the question if a population of this rodent is also present in the Island.

Within a more general study on the epidemiology of *Leptospira* in Italian wildlife, this paper reports the first case of *Leptospira* serovar Pomona isolation in *H. cristata*.

## 2 MATERIAL AND METHODS

### 2.1 Samples collection

In Autumn 2018, sampling was performed on two crested porcupines died for traumatic impact in a veterinary clinic, in the Grosseto province (Tuscany, Italy). The first one was recovered in the area of Pescia Fiorentina (Capalbio), whereas the second one in the area of Arcille (Campagnatico). At the clinic, animals were treated with enrofloxacin and dexamethasone for about 3 days before the death. From each carcass, during necropsy, kidneys and a blood sample from the heart cavity were collected. Before the necropsy, both animals were sexed and weighted and the age class was estimated (porcupette < 5 kg; 5 kg ≤ sub‐adult < 11 kg; adult ≥ 12 kg; (Coppola, Vecchio, & Felicioli, 2019).

### 2.2 Microscopic agglutination test

Blood samples were centrifugated at 1,200 *g* rpm for 10 min to obtain the serum. The sera were tested to detect *Leptospira* antibodies by MAT (OIE, 2018). Titre of 1:100 was considered as positive. The *Leptospira* live antigens used for MAT were as follows: Icterohaemorrhagiae (serogroup Icterohaemorrhagiae, strain Bianchi), Canicola (serogroup Canicola, strain Alarik), Pomona (serogroup Pomona, strain Mezzano), Grippotyphosa (serogroup Grippotyphosa, strain Moskva V), Tarassovi (serogroup Tarassovi, strain Mitis Johnson), Bratislava (serogroup Australis, strain Riccio 2), Hardjo (serogroup Sejroe, serovar Hardjoprajitno), Castellonis (serogroup Ballum, strain Castellon 3), Copenhageni (serogroup Icterohaemorrhagiae, strain Wijmberg), Bataviae (serogroup Bataviae, strain Pavia), Australis (serogroup Australis, strain Ballico), Zanoni (serogroup Pyrogenes, strain Zanoni), Saxkoebing (serogroup Sejroe, strain Mus 24), Sejroe (serogroup Sejroe, strain Topo 1), Poi (serogroup Javanica, strain Poi), Mini (serogroup Mini, strain Sari), Lora (serogroup Australis, strain Riccio 37), Hardjo (serogroup Sejroe, strain Farina), Autumnalis (serogroup Autumnalis, strain Akiyami A) and Hebdomadis (serogroup Hebdomadis, strain Hebdomadis).

### 2.3 *Leptospira* spp. isolation

The kidney samples were cultured in Ellinghausen–McCullough–Johnson–Harris (EMJH) medium (Difco). A portion of 10 cm^3^ from each porcupine kidney was homogenized with 5 ml of sterile water. One ml of homogenate was cultured in 5 ml of EMJH, incubated at 30 ± 1°C for 120 days and checked every 10 days under dark‐filed microscopy to assess bacterial growth.

In case of positive cultures, subcultures were performed to maintain the isolated strains alive.

### 2.4 *Leptospira* spp. genotyping

Isolated *Leptospira* were genotyped using a multilocus sequence typing scheme encompassing housekeeping genes (Ahmed et al., 2006; Boonsilp et al., 2013; Varni et al., 2014).

The amplification of each target gene was performed with HotStarTaq Master Mix Kit (Qiagen). Amplicons were further sequenced (BMR Genomics) using the same primer sets and analysed using BioEdit Software (Hall, 1999).

### 2.5 Molecular analysis

DNA was extracted from each kidney using the Quick‐DNA Plus Kits (Zymo Research) according to the manufacturer's instructions.

The *lipL32* gene Taqman RealTime PCR was performed on a Rotorgene Corbett 6000 (Corbett Research) to detect pathogenic leptospires (Stoddard, Gee, Wilkins, McCaustland, & Hoffmaster, 2009). The following thermal conditions were employed: a holding stage of 95°C for 5 min, and 45 cycles of 95°C for 15 s and 60°C for 30 s. Samples with Ct *lipL32*<35 were considered as positive.

### 2.6 Histopathology and immunohistochemistry

Representative portions of the kidneys collected during necropsy were routinely processed, paraffin‐embedded and 5‐µm‐thick sections were stained with haematoxylin and eosin, Masson trichrome Goldner and Warthin Starry stains. Tissue sections were also submitted to immunohistochemistry. Antigen retrieval was achieved on the slides by placing them in a bath of 10 mmol/L citric acid (pH 6) and boiling for 16 min in an 800‐W microwave oven. The slides were dried at room temperature and washed with running tap water. A peroxidase block was performed, and the slides were incubated with specific rabbit antisera against *Leptospira interrogans* serogroup Pomona and *Leptospira kirschneri* serogroup Grippotyphosa. The primary antibodies were diluted 1:300 in a buffer solution (PBS) prior to incubation. A polyclonal horse serum (1 drop diluted in 1 ml of PBS) was used as a secondary antibody for 30 min at room temperature (Vector Laboratories). Antibody binding was detected using a streptavidin‐biotin‐peroxidase kit (Vector Laboratories). The enzymatic reaction was developed with the use of 3‐1‐diaminobenzydine (Sigma Chemical) as a substrate. Stained slides were subsequently counter‐stained in haematoxylin for 40 s followed by a wash in tap water, dehydration in graded alcohols (70%, 90% and 100%), and clearance with xylene. Sections were mounted in DPX (08600E; Surgipath Europe). As a positive control, a pig kidney culture‐positive for serogroup Pomona and a mouse kidney culture‐positive for serogroup Grippotyphosa were used.

## 3 RESULTS

**FIGURE 1:**
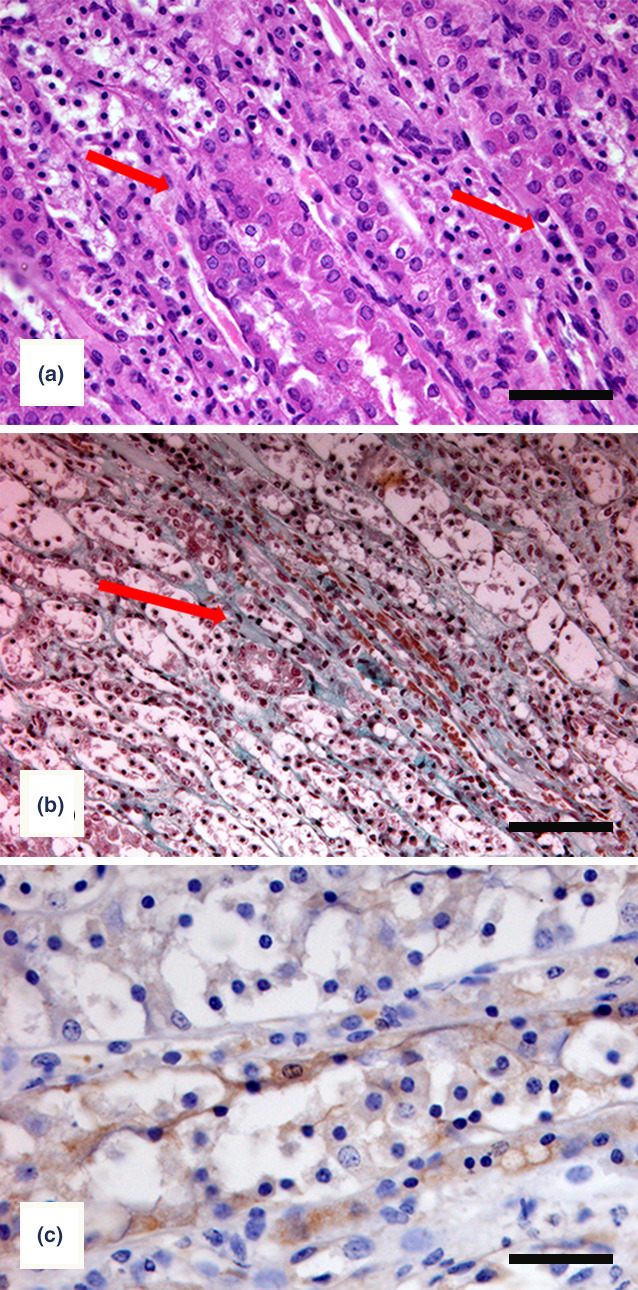
Crested porcupine kidney. Renal alterations associated with *Leptospira* infection. (a) Mild interstitial nephritis characterized by lymphocyte and plasma cell infiltration (arrows; H‐E; bar = 50 μm). (b) Mild interstitial fibrosis (arrow) surrounded by scattered inflammatory cells (Masson trichrome Goldner stain; bar = 50 μm). (c) Leptospiral antigen is present within tubular epithelial cells undergoing regressive changes (immunohistochemical staining using anti serovar Pomona antiserum and haematoxylin counterstain; bar = 30 μm)

Both exanimate porcupines were female, one adult of 11 kg (more than 1 year old) and a porcupette (around 2 months old) of 1.4 kg. The kidney of the porcupette showed small gray‐white focal lesions, mainly located in the renal cortex and varying from 1 to 2 mm in diameter. Microscopically, a mild chronic interstitial nephritis was present, characterized by vacuolar degeneration of the tubular epithelium and scattered interstitial foci consisting of lymphocytes and plasma cells (Figure 1a), accompanied by interstitial fibrosis (Figure 1b). In silver‐stained sections, leptospires were never detected in the tubular lumen adhering to the luminal surface of tubular cells, whereas Intracytoplasmic spherical bodies within cells of a tubule undergoing regressive changes were observed. In immunoperoxidase‐stained sections, using antisera against *Leptospira* serogroup Pomona an intense immunoreactivity for leptospiral antigen was detectable within the tubular epithelia cells and in cellular debris in tubular lumen (Figure 1c), whereas the absence of immune‐labelling was observed when the antiserum against *Leptospira* serogroup *Grippotyphosa* was used.

Both sera samples resulted negative to MAT for all *Leptospira* serogroups tested. Only in porcupette kidney, *Leptospira* DNA was detected and after 2 months of incubation, the bacterium was isolated. The isolated strain was identified as *L. interrogans* serogroup Pomona serovar Pomona, showing the sequence type (ST) 140 for scheme 1, ST 4 for scheme 2 and ST 58 for scheme 3.

## 4 DISCUSSION

Only recently, crested porcupine was investigated as potential vector or host for a wide range of parasites and micro‐organisms, such as fleas and hard ticks (Mori et al., 2015; Scaravelli, Senini, & Bonacci, 2017), *Giardia duodenalis* (Coppola, Maestrini, et al., 2020) and pathogenic *Leptospira* (Coppola, Cilia, et al., 2020). For the first time, *L. interrogans* serogroup Pomona serovar Pomona was isolated from one out of two analysed porcupine kidneys. Both sampled sera were negative for *Leptospira* serovars Pomona and for the other *Leptospira* serovars tested, whereas pathogenic *Leptospira* DNA was detected only in renal tissue from porcupette. The seronegativity of *Leptospira*‐positive subjects was previously reported for other species (Agampodi, Matthias, Moreno, & Vinetz, 2012; Hall & Lambourne, 2014; Merien, Baranton, & Perolat, 1995) and for porcupine (*S. villosus*), as well (Fornazari et al., 2018). The serological negativity of *Leptospira*‐positive porcupette could be related to the quality of blood sample collected from the heart cavity or to an early or chronic infection. However, a chronic infection seems to be unlikely due to the young age of the animal. At the same time, the presence of an early stage infection can be excluded considering the stage of renal lesions, characterized by the presence of inflammatory infiltrates, mild fibrosis and the absence of a large amount of leptospires localized in tubular lumen, typically detectable during early *Leptospira* infection. At this stage, the micro‐organisms should be numerous, intact and easy to visualize by both the immunohistochemical and silver‐staining methods and the perifocal inflammatory infiltrates are scanty or absent (Michna & Campbell, 1969). Subsequently, when the inflammatory cells surround the infected tubules, leptospires are lysed, clumped and leptospiral antigen are taken up by tubular cells. In this case, immunohistochemical studies allow to detect the presence of *Leptospira* antigen in tubular epithelial cells and to reveal intracytoplasmic spherical bodies within the cells of renal tubules undergoing regressive changes, as previously described in leptospiral nephritis in swine (Scanziani, Sironi, & Mandelli, 1989). Previous electron microscopic studies demonstrated the presence of degenerating leptospires within these vesicles in tubular cells (Ellis, Robertson, Hustas, & Kirby, 1983).

The seronegativity of *Leptospira*‐positive subject could be related to the antibiotic treatment with enrofloxacin, performed during the hospitalization. Activity of enrofloxacin against *Leptospira* is documented in vitro, but some studies showed increased MIC values in recent isolates (Liegeon, Delory, & Picardeau, 2018; Moreno et al. 2016). Carrascosa et al. (2017) showed a low effectiveness of this antibiotic in vivo in order to prevent *Leptospira* renal colonization in hamster and a decrease in antibiotic effectiveness when the treatment is delayed from the starting of the infection. However, antibiotic treatment could have affect the antibodies response, leading to negative MAT results, as previously reported by Ricaldi, Swancutt, and Matthias (2013) and Courdurie et al. (2017). The antibiotic treatment could have also determined the lack of leptospires in the renal tissue highlighted with specific Warthin Starry staining. The reduced bacterial load has also been highlighted by the long incubation period of the culture before the isolation of the *Leptospira* strain (Azizi, Kheirandish, & Rahimi, 2014).

Rodents are well known important *Leptospira* carriers, involved in the infection transmission to animals and humans (Blasdell, Morand, Perera, & Firth, 2019; Mori et al., 2017). The evidence of a possible *Leptospira* infection in crested porcupine was previously investigated in the same studied area. Seven out of 14 (50%) of porcupine sera resulted positive to anti‐*Leptospira* antibodies detection; Icterohaemorrhagiae resulted the most prevalent serogroup (4 positive sera), followed by serogroup Pomona and Australis (2 sera, respectively). Titres of 1:400 and 1:100 were recorded for serogroup Pomona (Coppola, Cilia, et al., 2020). At the best of Authors knowledge, excluding *H. cristata,* among the Hystricomorpha rodents, isolation of *Leptospira* serovar Pomona from kidney, blood and urine was only performed in one North American porcupine (Mitchell et al., 1966) and no other *Leptospira* serovar were isolated. The crested porcupine *Leptospira* serovar Pomona infection documented in this paper could be the result of habitat sharing with wild boar (*Sus scrofa*) which are present in Tuscany (Italy) with an high‐density population (Massei et al., 2015; Santilli & Varuzza, 2013). The wild boar plays a key role in the spreading of some *Leptospira* serovars, such as Pomona, in the environment (Bertelloni et al., 2019; Chiari et al., 2016). These factors could strongly increase and promote the possibility of porcupine infection.

The isolation of *Leptospira* from crested porcupine suggests that *H. cristata* could be a new potential natural host for this bacterium.

Authors are aware of the limit of the study, since it represents a case report of *Leptospira* serovar Pomona infection in crested porcupine. Despite the single positive sample, this result could be a useful contribute to the description of the epidemiology of leptospirosis. Further investigations are needed to specifically determine the role of crested porcupine as accidental or as maintenance host for *Leptospira*.

## 5 CONCLUSION

Leptospirosis is one of the most widespread and emerging zoonotic disease in the world, and wild animals were known to be *reservoir* of *Leptospira*. The results obtained in this paper show that *H. cristata* could be a *Leptospira* host, as hypothesized for other hystricomorph rodents. The infection by serovars Pomona, typically observed in swine and wild boar, could suggest an adaptability and/or a change in host range for this serovar, as assumed for other serovars. Further investigations are needed to clarify the role of this peculiar rodent in the epidemiology of leptospirosis in Italy.

## CONFLICT OF INTEREST

All authors declare no conflict of interests.

## AUTHOR CONTRIBUTION

**Giovanni Cilia**: Data curation; Formal analysis; Investigation; Methodology; Writing‐original draft; Writing‐review & editing. **Fabrizio Bertelloni**: Data curation; Formal analysis; Methodology; Writing‐original draft; Writing‐review & editing. **FRANCESCA COPPOLA**: Data curation; Investigation; Writing‐original draft; Writing‐review & editing. **Barbara Turchi**: Data curation; Investigation; Methodology; Writing‐review & editing. **Claudia Biliotti**: Data curation; Investigation; Writing‐review & editing. **Alessandro Poli**: Data curation; Investigation; Methodology; Writing‐review & editing. **Francesca Parisi**: Data curation; Investigation; Methodology; Writing‐review & editing. **Antonio Felicioli**: Conceptualization; Data curation; Supervision; Writing‐original draft; Writing‐review & editing. **Domenico Cerri**: Conceptualization; Data curation; Resources; Supervision; Writing‐review & editing. **Filippo Fratini**: Conceptualization; Data curation; Investigation; Methodology; Resources; Supervision; Writing‐original draft; Writing‐review & editing.

## ETHICAL STATEMENT

The authors confirm that the ethical policies of the journal, as noted on the journal's author guidelines page, have been adhered to. No ethical approval was required.

## Data Availability

The data that support the findings of this study are available from the corresponding author upon reasonable request.
